# *CDKN2A/B* mutations and allele-specific alterations stratify survival outcomes in IDH-mutant astrocytomas

**DOI:** 10.1007/s00401-023-02639-0

**Published:** 2023-10-13

**Authors:** Richard A. Hickman, Erika Gedvilaite, Ryan Ptashkin, Anne S. Reiner, Robert Cimera, Subhiksha Nandakumar, Adam Price, Chad Vanderbilt, Tara Fahy, Robert J. Young, Alexandra M. Miller, Ingo K. Mellinghoff, Marc K. Rosenblum, Marc Ladanyi, Maria E. Arcila, Yanming Zhang, A. Rose Brannon, Tejus A. Bale

**Affiliations:** 1 Human Oncology and Pathogenesis Program, Sloan Kettering Institute, New York, NY 10065 USA; 2 Department of Pathology, Memorial Sloan Kettering Cancer Center, New York, NY 10065 USA; 3 Murtha Cancer Center Research Program, Uniformed Services of the Health Sciences, Bethesda, MD 20817 USA; 4 Henry M. Jackson Foundation for the Advancement of Military Medicine, Inc., Bethesda, MD 20817 USA; 5 Department of Epidemiology and Biostatistics, Memorial Sloan Kettering Cancer Center, New York, NY 10065 USA; 6 Department of Radiology, Memorial Sloan Kettering Cancer Center, New York, NY 10065 USA; 7 Department of Neurology, Memorial Sloan Kettering Cancer Center, New York, NY 10065 USA

IDH-mutant astrocytomas (IDHA) are initially slow-growing tumors that almost inevitably progress [4]. Homozygous deletion (HOMDEL) of *CDKN2A* and/or *CDKN2B* (*CDKN2A/B*), now a WHO grade 4 defining criterion for IDHA, is associated with rapid progression and poor overall survival (OS) [1, 9]. We examined the prognostic significance of additional *CDKN2A/B* inactivating events, including nonsynonymous mutations and allele-specific copy number alterations (ASCNA) by analyzing 347 prospectively tumor-matched normal sequenced IDHA (347 patients, supplemental table 1) using FACETS (Fraction and Allele-Specific Copy Number Estimates from Tumor Sequencing), a SNP-based algorithm to assess ASCNA across genomic targets [8], with validation using The Cancer Genome Atlas (TCGA, 188 tumors/patients, supplemental methods).

*CDKN2A/B* alterations were found in up to 15% of IDHA (Fig. 1a, b): 12% exhibited *CDKN2A/B* loss (n = 42/347) and were associated with higher histologic grade (supplemental table 2). *CDKN2A/B* loss, *PDGFRA* gain, and *CDK4* gain associated with shorter OS by multivariable Cox proportional hazards modelling (supplemental Fig. 1e). Nonsynonymous mutations in *CDKN2A* were uncommon (n = 9, 2.6%), but were mostly classified as oncogenic or likely oncogenic by OncoKB™ (n = 6, Fig. 1b, supplemental table 3) [2]. *CDKN2A*-mutant tumors were higher grade and had shortened OS (median OS: 1.6 years, 95% CI: 0.8 years–not reached [NR]) versus non-mutant tumors (median OS: 12.6 years, 95% CI: 11.4 years–NR, *P* < 0.001, Fig. 1c), approximating the OS of tumors with *CDKN2A/B* copy loss (median survival: 3.0 years, CI: 1.4 years–NR) even after excluding hypermutant tumors [10].

**Fig. 1:**
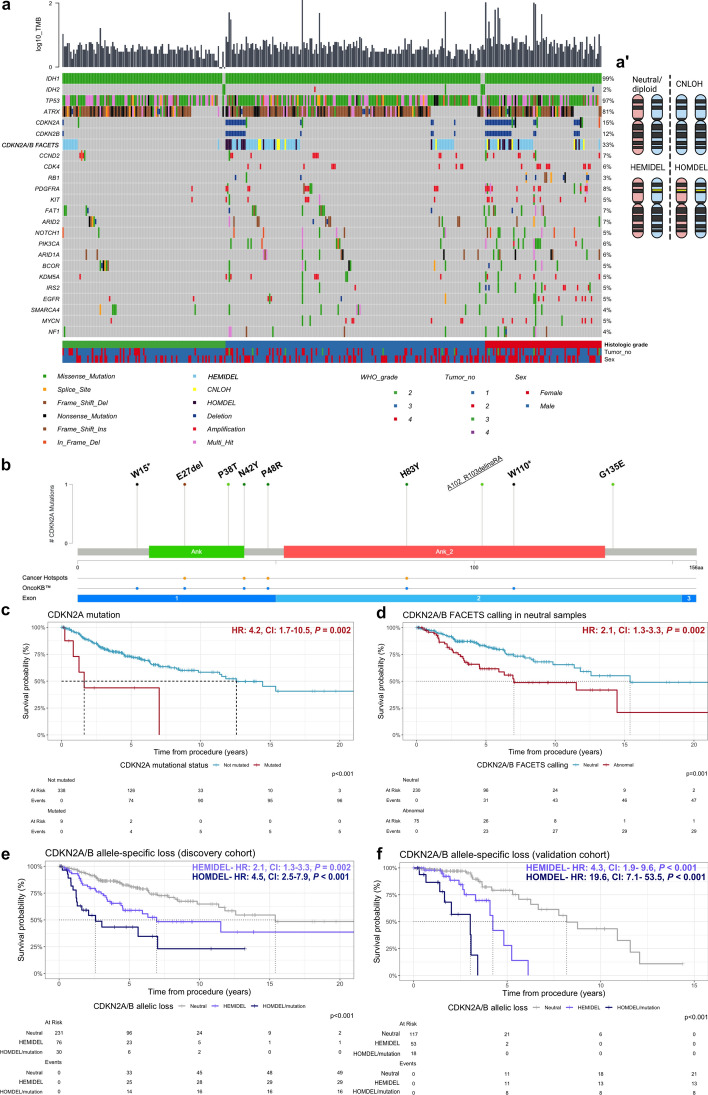
**a** Oncoplot depicting frequently altered genes and log_10_ tumor mutational burden (TMB) in 347 IDHA. **a**' CDKN2A/B allele-specific copy number states at 9p21.3. Yellow band = CDKN2A/B deletion, red and blue shades discern parental alleles. **b** Lollipop plots display variants in copy neutral CDKN2A/B (underlined: hypermutant). **c** Nonsynonymous CDKN2A mutations shorten OS. **d** ASCNA in CDKN2A/B copy neutral samples conferred worse OS than FACETS neutral. **e** CDKN2A/B HEMIDEL (n = 76) confers intermediate OS between patients with CDKN2A/B HOMDEL/mutation and CDKN2A/B neutral/without mutation. **f** TCGA validation cohort; HR—hazard ratios, CI—confidence intervals, P values within graphs refer to univariable Cox-proportional hazards regression models, log-rank test

FACETS detected hemizygous deletion (HEMIDEL) in 23% of IDHA (n = 81/347), HOMDEL in 6% (n = 21), and copy-neutral loss of heterozygosity (CNLOH) in 3% (n = 11), mostly in high histologic grade tumors (supplemental table 4). The remaining 67% (n = 234) were neutral (supplemental Fig. 2a). Of 305 copy neutral tumors, FACETS detected ASCNA in 75 (25%) demonstrating shorter OS (median 7.0 years, 95% CI: 5.9 years–NR) vs. FACETS neutral (15.4 years, 95% CI: 11.8 years–NR, *P* = 0.0014, Fig. 1d). HEMIDEL IDHA had shorter OS than neutral (median survival: 6.9 years (95% CI: 4.5 years–NR) vs. 15.4 years (95% CI: 11.8 years–NR, *P* < 0.001), but longer OS than HOMDEL/mutant cases (median survival: 2.6 years, 95% CI: 1.3 years–NR, *P* = 0.018, Fig. 1e). CNLOH of *CDKN2A/B* portended poor prognosis (median survival: 2.3 years, 95% CI: 1.6 years–NR, *P* < 0.001, supplemental Fig. 2b). We verified intermediate OS of *CDKN2A/B* HEMIDEL between *CDKN2A/B* HOMDEL/mutant and neutral tumors (Fig. 1f) in the TCGA cohort (supplemental methods). Gains of *CDK4* and/or *CCND2* worsened OS in *CDKN2A/B* HEMIDEL tumors (median survival: 3.2 years (95% CI: 1.4 years–NR) versus 11.5 years (95% CI: 4.5 years-NR, *P* = 0.011, supplemental Fig. 2c, d).

Matched tumor-normal NGS with FACETS analysis detects a range of prognostically relevant alterations in *CDKN2A*/*B* and other genes. FACETS showed good concordance with SNP-microarray in a subset of cases (supplemental Fig. 3). Kocakavuk et al. recently described shortened OS across multiple cohorts of IDH-mutant, 1p/19q intact gliomas with *CDKN2A* HEMIDEL using copy number profiles from NGS/methylation data [3]. These findings emphasize the clinical relevance of detection of *CDKN2A/B* HEMIDEL in IDHA. [6, 7]. As a molecularly-targeted therapeutic has shown promise in IDH-mutant grade 2 gliomas [5], extending the utility of NGS with bioinformatic tools like FACETS may help to both improve patient management and guide optimal treatment in the future.

### Supplementary Information

Below is the link to the electronic supplementary material.

Supplementary file1 (DOCX 25 kb)

Supplementary file2 (DOCX 17041 kb)

## Data Availability

The datasets analyzed in the current study are available from the corresponding author on reasonable request.
