# Supplementary material for: CDKN2A/B mutations and allele-specific alterations stratify survival outcomes in IDH-mutant astrocytomas
Source: Acta Neuropathol. 2023 Oct 13;146(6):845–7. doi: 10.1007/s00401-023-02639-0 (PMC10628020; doi:10.1007/s00401-023-02639-0)
Supplement: Supplementary file 1 — Supplementary file1 (DOCX 25 kb) [file 401_2023_2639_MOESM1_ESM.docx]

**Supplementary materials and methods**

**Ethical approval**

This study was conducted in accordance with the Declaration of Helsinki and was approved by the Institutional Review Board at Memorial Sloan Kettering Cancer Center. This protocol allows for clinicopathologic and molecular data retrieval from patients who provided informed consent or a waiver of consent.

**Discovery cohort- MSKCC**

The MSK-IMPACT™ database was retrospectively searched for neuroepithelial tumors with *IDH1* or *IDH2* mutations that were not designated oligodendrogliomas. Of 463 tumor samples retrieved, cases were excluded if *IDH1*/*IDH2* mutations were non-hotspot, cerebrospinal fluid samples, and if 1p/19q codeletion was detected by SCNA or fluorescence in-situ hybridization. Three-hundred and eighty-two suitable tumor samples from 347 patients were then identified for this study. When multiple samples were available for an individual patient, the earliest sample was selected and later samples were excluded, leaving 347 samples for study. Samples included both newly diagnosed and recurrent tumors. The diagnosis of IDHA was then confirmed by a board-certified pathologist subspecialized in neuropathology and molecular genetic pathology.

*Sample preparation*

Samples submitted for routine NGS were initially selected by a pathologist. A representative hematoxylin and eosin-stained slide was examined to assess for tumor purity; all cases had an estimated tumor purity >10% for sequencing and macro-dissection was performed to enrich the tumor fraction where appropriate. Each sample had a corresponding patient-matched blood sample as a patient-specific normal control. DNA was extracted using standard validated protocols.

*Genetic analysis*

Comprehensive sequencing was performed by MSK-IMPACT™, an FDA-authorized hybridization capture-based targeted next generation sequencing (NGS) assay that enables deep targeted sequencing to detect somatic genetic alterations in human specimens (previously described by Cheng *et al.* [2]). The analysis pipeline incorporates tumor-matched normal sequencing for the identification of somatic alterations specific to the tumor tissue as well as exclusion of variants that may be due to clonal hematopoiesis. Furthermore, the incorporation of a matched normal sample enables identification of parental SNPs that can be used in determination of ASCNA within the tumor. Briefly, custom capture probes that target all protein-coding exons and select intronic regions of 341 to 505 cancer-related genes were designed (31 samples submitted for version 3 (341 genes), 74 samples submitted for version 5 (410 genes), 207 samples submitted for version 6 (468 genes) and 35 samples submitted for version 7 (505 genes). All versions incorporated *CDKN2A*, *CDKN2B*, *CDK4*, *CCND2*, and *PDGFRA.* The standard DNA input was 250 ng but could be lowered to 50 ng if the sample was limited. Captured libraries of both tumor and normal were sequenced on Illumina HiSeq 2500 or NovaSeq 6000 instruments. Point mutations and indels were detected using MuTect, Vardict and SomaticIndelDetector. SCNA were detected by comparing loess-normalized sequence coverage of targeted loci of the tumor with a standard diploid non-tumor sample. *CDKN2A/B* loss by SCNA referred to either *CDKN2A* and/or *CDKN2B* showing significant copy loss. Structural variants (SV) were identified using DELLY [4]. Variants were annotated using VEP/ ANNOVAR; variants that were considered germline or due to clonal hematopoiesis in the patient-matched blood normal sample were filtered out. All variants were reviewed and called by a bioinformatic analyst and a board-certified molecular genetic pathologist. A total of 2512 somatic nonsynonymous mutations, 793 somatic copy number alterations (SCNA), and 58 structural rearrangements were detected across 347 IDHA (figure 1a). Nearly all *IDH1/2* nonsynonymous driver mutations occurred within *IDH1* (n=343, 99%) and 289 (84%) were *R132H*. Tumor mutational burden (TMB) was calculated as mutations per megabase (Mb) utilizing only nonsynonymous coding mutations in the calculations (including frameshift, point mutations, and small insertions and deletions (indels)). Cases with ≥13.8 nonsynonymous mutations/Mb were considered hypermutant. Fraction of genome altered (FGA) was downloaded for each sample using cBioPortal and refers to the proportion of the genome affected by copy number gains or losses [1].

**FACETS**

Allele-specific copy number analysis was performed using FACETS [5]. This algorithm requires tumor-matched normal sequencing to avoid mapping biases; all tumors had matched normal samples. Allelic imbalance (loss of heterozygosity (LOH)) was assessed using the allele log-odds-ratio (logOR), which compares the variant allele read count of heterozygous single nucleotide polymorphism (SNP) sites in the tumor with that in the normal tissue. The diploid state (diplogR) was manually reviewed by bioinformaticians (EG/RP) and optimized according to diploid chromosomal states (total copy number (tcn) = 2, lesser/ minor copy number (lcn) = 1). HEMIDEL of *CDKN2A/B* referred to tcn:lcn 1:0 states in *CDKN2A* and/or *CDKN2B*, HOMDEL of *CDKN2A/B* referred to tcn:lcn 0:0 states in either *CDKN2A* and/or CDKN2B, CNLOH of *CDKN2A/B* referred to tcn:lcn 2:0 states in either *CDKN2A* and/or *CDKN2B*.

For the assessment of CDKN2A/B ASCNA and survival outcomes, tumors with *CDKN2A/B* mutation and/or *CDKN2A/B* HOMDEL were combined as a poor prognostic category and compared with *CDKN2A/B* HEMIDEL and neutral samples after excluding any remaining CNLOH cases. This analysis excluded patients with CNLOH of CDKN2A/B and lacked CDKN2A/B mutations (n = 10).

**Validation cohort- TCGA**

To validate our ASCNA findings in a separate cohort, we separately analyzed combined data from two independent TCGA cohorts (Brain Lower Grade Glioma (TCGA, PanCancer Atlas) and Glioblastoma Multiforme (TCGA, PanCancer Atlas)) using cBioPortal [1]. This combined dataset includes 1106 samples from 1099 patients. We identified IDH-mutant astrocytomas by selecting samples with a detailed cancer type of “Glioblastoma Multiforme”, “Astrocytoma”, “Oligoastrocytoma”, and “Lower Grade Glioma”, and excluded “Oligodendroglioma”, resulting in 917 samples from 910 patients. We restricted all remaining samples to those that harbored *IDH1* or *IDH2* mutations, *TP53* and/or *ATRX* mutations, and were 1p/19q intact, leaving 224 samples from 224 patients. For the assessment of CDKN2A/B ASCNA and survival outcomes, we further excluded samples that had *CDKN2A/B* copy gains, CNLOH, and those without calls for both *CDKN2A* and *CDKN2B*, leaving 188 samples for this assessment.

**SNP microarray**

SNP microarrays with 2.67 million probes, including 750000 common and rare SNP probes (Cytoscan, Affymetrix, Santa Clara, CA), were performed on selected tumors with ASCNA following the manufacturers’ protocol. Data analysis was performed using Affymetrix ChAS 4 software.

**Statistical analysis**

Follow-up time for a sample/ patient was defined as the duration of time from surgical procedure until death (event) or last follow-up (right censored). OS was described using Kaplan-Meier methodology. The median follow-up time of survivors for the discovery cohort was 4.3 years (IQR: 4.4 years, n = 119). The log-rank test was performed to assess differences in OS across defined groups and hazard ratios (HR) with corresponding 95% confidence intervals (CI) were estimated using univariable Cox proportional hazards regression modeling. A multivariable Cox proportional hazards model was also built to estimate HR in an adjusted setting. Data analysis was performed using R version 4.1.0, with the following packages: ggplot2, survival, survminer, ggsurvfit, gtsummary, flipPlots, and maftools (version 2.10.05). Genes with somatic alterations (≥ 3% gene frequency) were visualized using the oncoplot function of maftools and gene enrichment analysis of high versus low histologic grade IDHA was performed using the clinicalEnrichment function of maftools (*P* < 0.05) [3]. SCNA groups were compared across WHO groups using the Chi-squared and Fisher’s tests as appropriate.

**References**

1 Cerami E, Gao J, Dogrusoz U, Gross BE, Sumer SO, Aksoy BA, Jacobsen A, Byrne CJ, Heuer ML, Larsson E (2012) The cBio cancer genomics portal: an open platform for exploring multidimensional cancer genomics data. Cancer discovery 2: 401-404

2 Cheng DT, Mitchell TN, Zehir A, Shah RH, Benayed R, Syed A, Chandramohan R, Liu ZY, Won HH, Scott SNet al (2015) Memorial Sloan Kettering-Integrated Mutation Profiling of Actionable Cancer Targets (MSK-IMPACT): A Hybridization Capture-Based Next-Generation Sequencing Clinical Assay for Solid Tumor Molecular Oncology. J Mol Diagn 17: 251-264 Doi 10.1016/j.jmoldx.2014.12.006

3 Mayakonda A, Lin D-C, Assenov Y, Plass C, Koeffler HP (2018) Maftools: efficient and comprehensive analysis of somatic variants in cancer. Genome research 28: 1747-1756

4 Rausch T, Zichner T, Schlattl A, Stütz AM, Benes V, Korbel JO (2012) DELLY: structural variant discovery by integrated paired-end and split-read analysis. Bioinformatics 28: i333-i339 Doi 10.1093/bioinformatics/bts378

5 Shen R, Seshan VE (2016) FACETS: allele-specific copy number and clonal heterogeneity analysis tool for high-throughput DNA sequencing. Nucleic acids research 44: e131-e131

**Acknowledgements**

This work was partly funded by the Marie-Josée and Henry R. Kravis Center for Molecular Oncology and the National Cancer Institute Cancer Center Core Grant No. P30-CA008748. The views expressed in this article are those of the authors and do not necessarily reflect the views or policies of the Uniformed Services University, the United States Department of Defense, the United States government, or the Henry M. Jackson Foundation for the Advancement of Military Medicine, Inc..
