# Supplementary material for: CDKN2A/B mutations and allele-specific alterations stratify survival outcomes in IDH-mutant astrocytomas
Source: Acta Neuropathol. 2023 Oct 13;146(6):845–7. doi: 10.1007/s00401-023-02639-0 (PMC10628020; doi:10.1007/s00401-023-02639-0)
Supplement: Supplementary file 2 — Supplementary file2 (DOCX 17041 kb) [file 401_2023_2639_MOESM2_ESM.docx]

**Supplemental tables and figures**

**
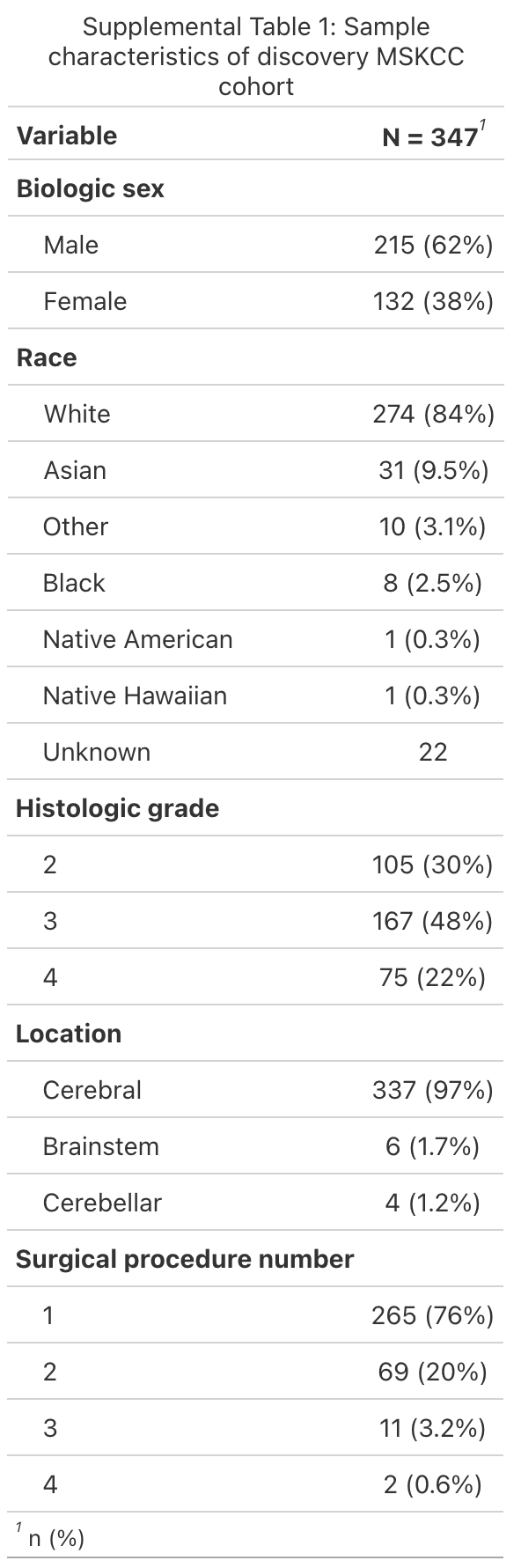
**

**
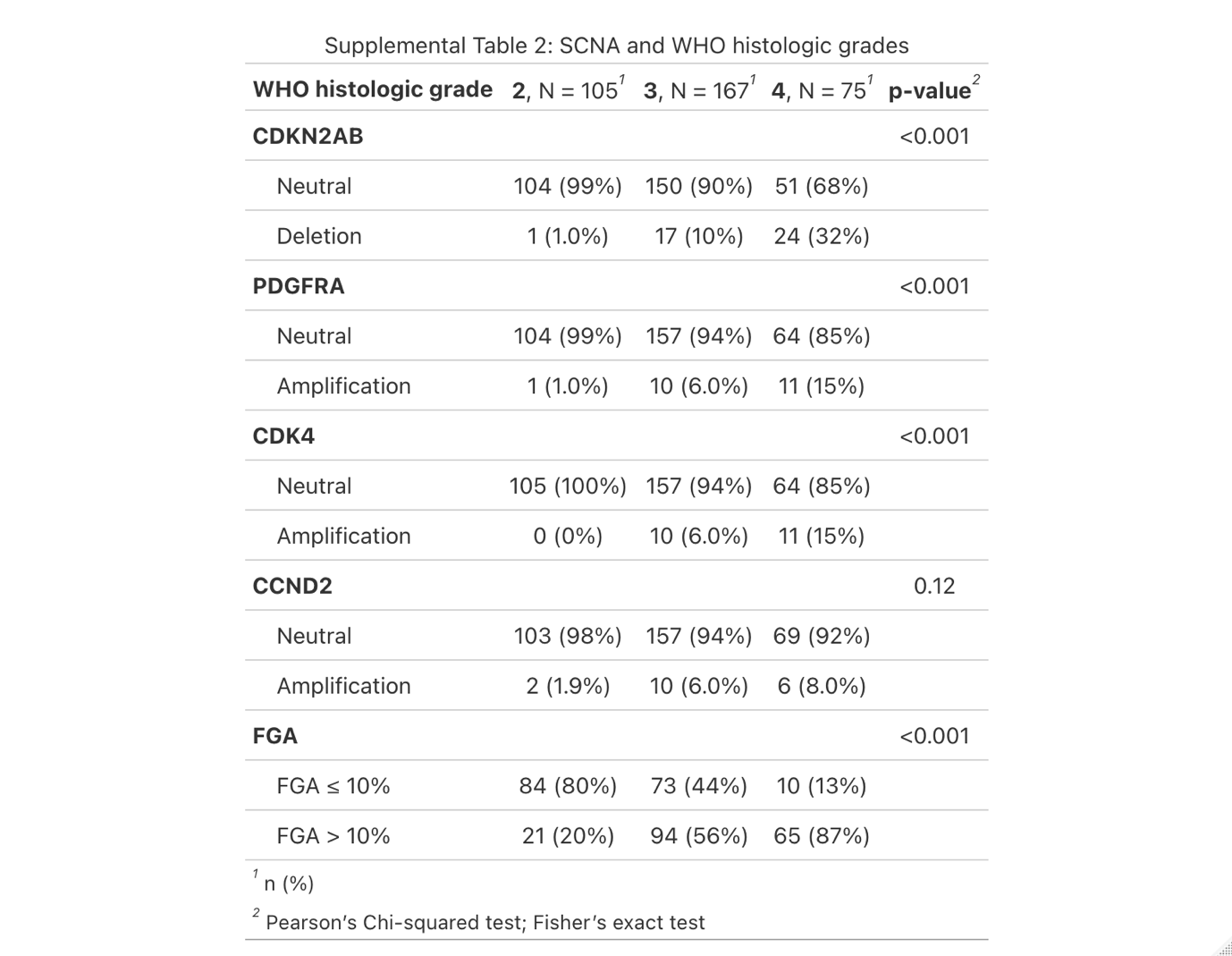
**

*Supplemental Table 2:* *12% of tumors exhibited CDKN2A/B loss (n=42/347) and were associated with higher histologic grade, as were tumors with gains of PDGFRA (6%, n=22), CDK4 (6%, n=21), and/or CCND2 (5%, n=18).*

| **Case #** | **WHO**  **histologic**  **grade** | **FACETS CDKN2A/B** | **Amino acid change** | ***CDKN2A* VAF** | ***IDH1* VAF** | **OncoKB** | **Status** | **Time (yrs)** | **TMB (mut/Mb)** |
| --- | --- | --- | --- | --- | --- | --- | --- | --- | --- |
| **1** | 4 | CNLOH | *P48R* | 0.97 | 0.22 | Likely oncogenic | Deceased | 0.84 | 9.1 |
| **2** | 4 | HEMIDEL | *W110** | 0.83 | 0.57 | Likely oncogenic | Alive | 5.24 | 6.1 |
| **3** | 3 | HEMIDEL | *G135E* | 0.79 | 0.47 | NA | Deceased | 7.01 | 8.9 |
| **4** | 4 | HEMIDEL | *N42Y* | 0.54 | 0.37 | Likely oncogenic | Deceased | 1.62 | 6.1 |
| **5** | 4 | HEMIDEL | *W15** | 0.31 | 0.18 | Likely oncogenic | Deceased | 1.25 | 7.9 |
| **6** | 4 | HEMIDEL | *E27del* | 0.24 | 0.25 | Likely oncogenic | Alive | 2.34 | 10.5 |
| **7** | 4 | Neutral | *P38T* | 0.10 | 0.42 | NA | Alive | 0.52 | 6.6 |
| **8** | 2 | Neutral | *H83Y* | 0.06 | 0.35 | Oncogenic | Alive | 0.14 | 4.9 |
| **9** | 3 | Neutral | *A102-R103delinsRA* | 0.13 | 0.34 | NA | Deceased | 0.23 | 125.5 |

*Supplemental Table 3: Profile of 9 tumors with CDKN2A mutations with associated variant allelic frequency (VAF), FACETS allele-specific copy number calls, and relationship to IDH1 VAF. OncoKB oncogenicity scores are also provided (NA- not assigned an OncoKB score). Sample 9 was hypermutated (≥13.8 nonsynonymous mutations/Mb). To exclude confounding bystander CDKN2A mutations in hypermutated samples, we re-analyzed OS after removing hypermutant cases, which upheld the worsened OS in the mutant group (median OS: 4.3 years (95% CI: 1.2 years–not reached) vs. 14.5 years (95% CI: 11.5 years–not reached), P=0.005, log-rank test). Three CDKN2A variants were present in the TCGA cohort and two of these (P48R and W110*) were also present in the discovery cohort and considered likely oncogenic by OncoKB.*

**
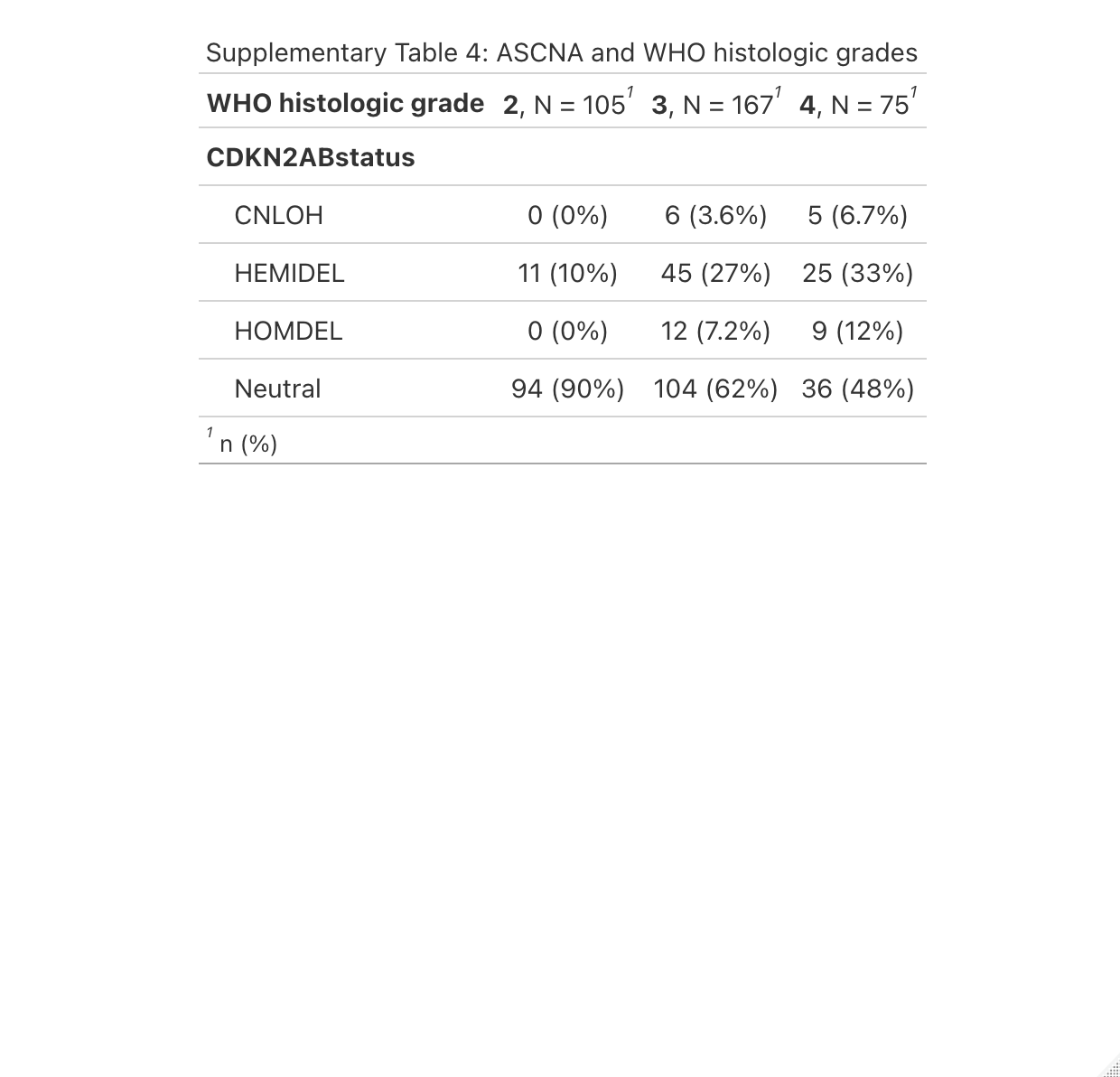
**

**
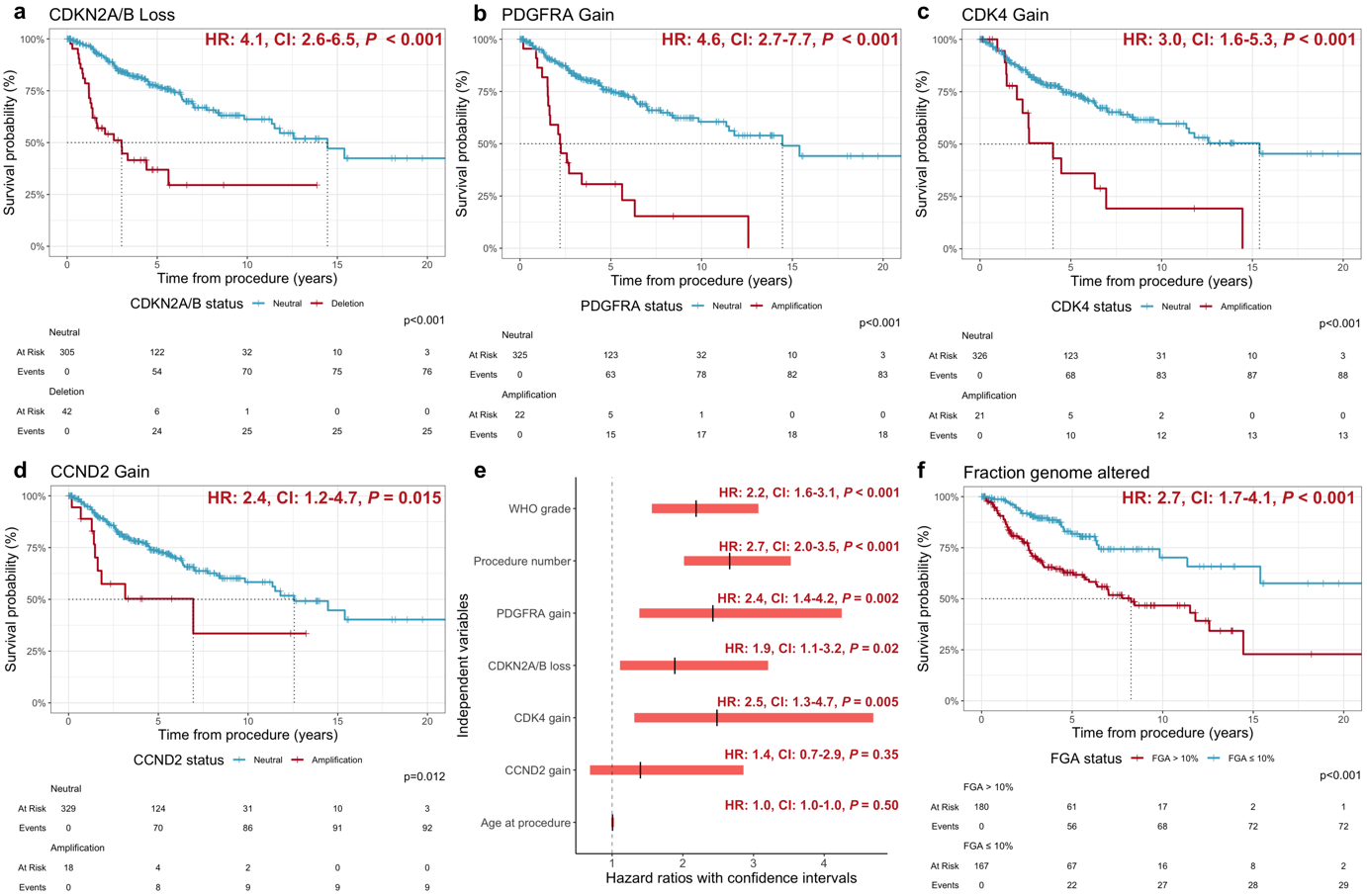
**

*Supplemental figure 1: Associations of the most frequent SCNA on OS in IDH-mutant astrocytomas from the discovery cohort (a-d). Multivariable analysis of associations of these SCNA and WHO grade, procedure number and age as independent variables. CDKN2A/B loss, PDGFRA gain, and CDK4 gain associated with shorter OS independent of WHO grade, age at procedure, or recurrent tumor status by multivariable Cox proportional hazards modelling (e). Fraction genome altered (FGA) > 10% associated with worsened OS than FGA ≤ 10% (f).*

*
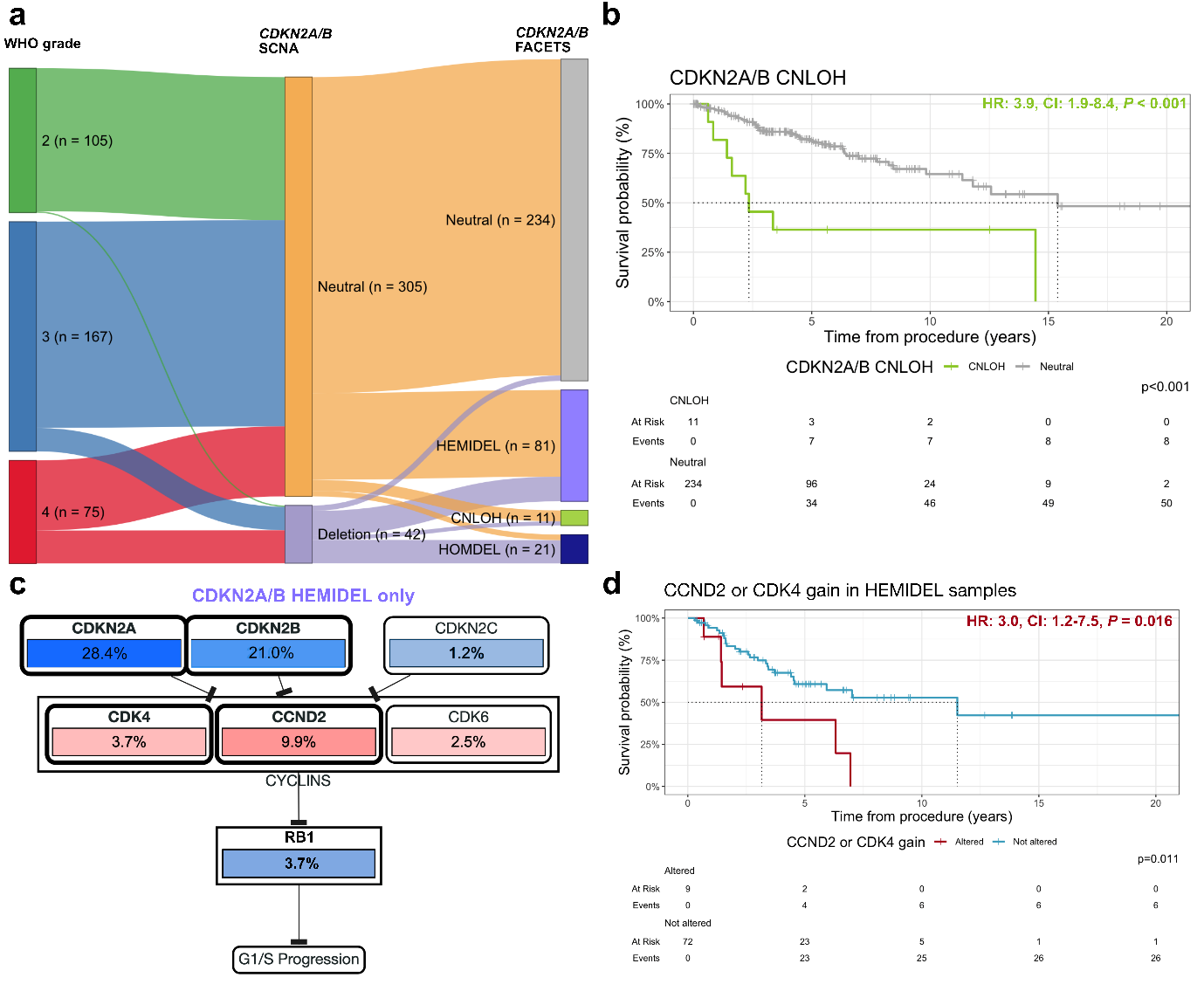
*

*Supplemental figure 2: a: Sankey plot illustrating the breakdown of cases by WHO histologic grade, CDKN2A/B SCNA, and allele-specific CDKN2A/B calls by FACETS in the discovery cohort. b: OS is worse in patients with CNLOH of CDKN2A/B than if neutral. c: Pathway Mapper showing the proportion of variants/ SCNA in genes of the cell cycle pathway of CDKN2A/B HEMIDEL only tumors. Note that CDKN2A and CDKN2B alterations refer to variants and SCNA not FACETS. The CDKN2A and CDKN2B genes encode for crucial tumor suppressor proteins of the cell cycle pathway by inhibiting CDK4 and cyclin D proteins. d: OS for samples with CDKN2A/B HEMIDEL by FACETS with additional SCNA copy gains in CCND2 and/or CDK4 have significantly worse OS than those without. HR- hazard ratios, CI- confidence intervals, P values within graphs refer to univariable Cox-proportional hazards regression models, log-rank test.*

*
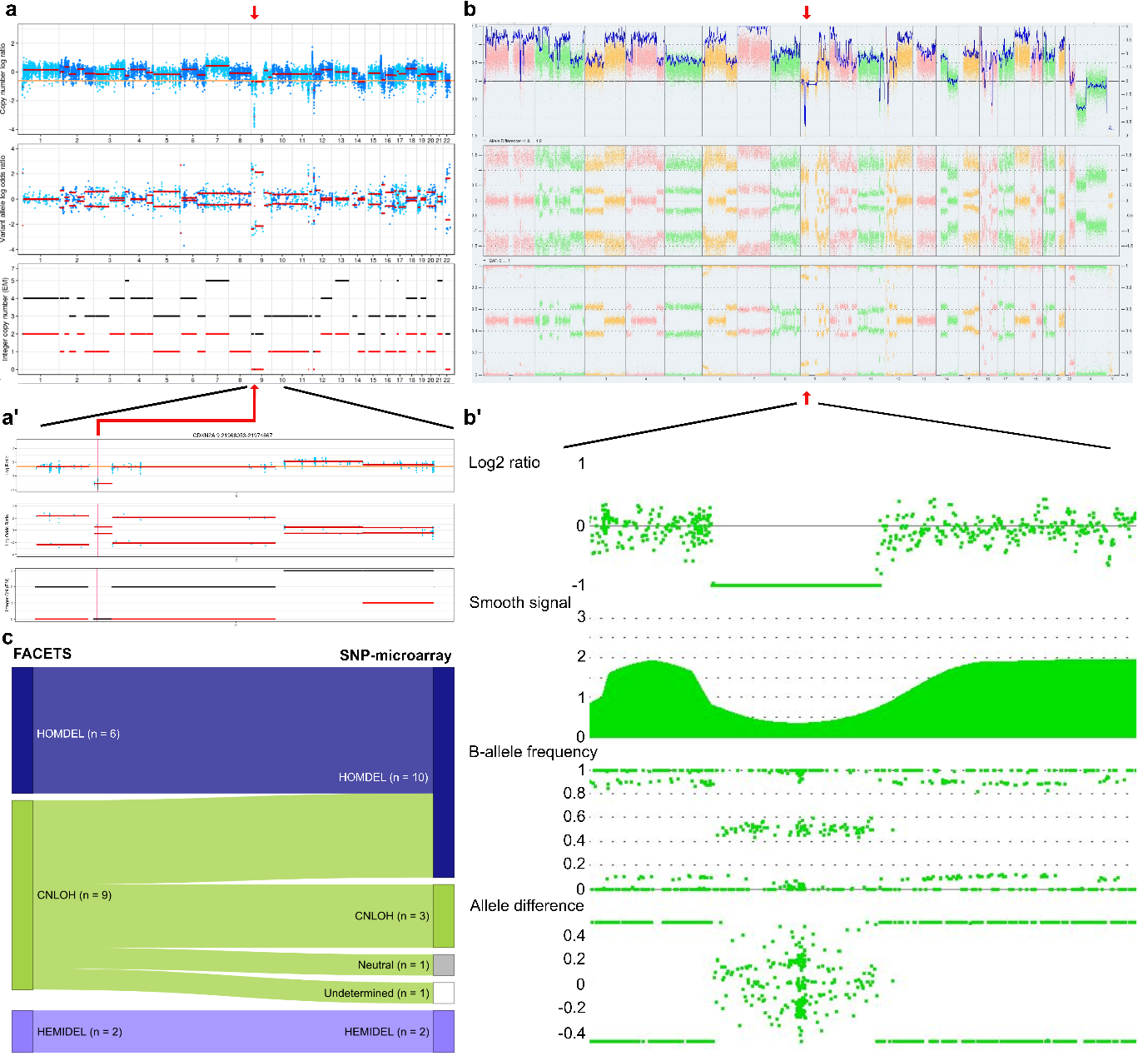
*

*Supplemental figure 3: Concordance analysis between FACETS calls for CDKN2A and SNP-microarray. a) FACETS plots for an IDHA with CDKN2A and CDKN2B HOMDEL (arrows indicate the location on 9p of the HOMDEL). Note that the diploid state of this sample was refitted according to 14q (horizontal orange line) and that the copy number log ratio refers to the initial fit. CDKN2A HOMDEL was detected before and after the refit. a') A zoomed in view of the CDKN2A region which is indicated by the vertical red line. b) SNP-microarray plot showing a whole genome view with high concordance and overlap of ASCNA with FACETS of the same case. Copy number log ratio is indicated on the upper left plot axis (b), smooth signal on the upper right axis, allele difference in the middle plot and B-allele frequency plots in the lower plot. The diploid state is seen at 14q. b') A zoomed in view of the CDKN2A region reveals the region of HOMDEL in CDKN2A. c) Sankey plot identifying the concordance and discordance between FACETS calls and SNP-microarray. CNLOH had the most discordance but in the majority indicated an ASCNA. Red arrows indicate the region of CDKN2A (a-b).*
